# Research on Automatic Classification and Detection of Mutton Multi-Parts Based on Swin-Transformer

**DOI:** 10.3390/foods12081642

**Published:** 2023-04-14

**Authors:** Shida Zhao, Zongchun Bai, Shucai Wang, Yue Gu

**Affiliations:** 1 Institute of Facilities and Equipment in Agriculture, Jiangsu Academy of Agricultural Sciences, Nanjing 210014, China; zsd@webmail.hzau.edu.cn; 2 College of Engineering, Huazhong Agricultural University, Wuhan 430070, China

**Keywords:** mutton processing, computer vision, deep learning, classification, detection, livestock meat

## Abstract

In order to realize the real-time classification and detection of mutton multi-part, this paper proposes a mutton multi-part classification and detection method based on the Swin-Transformer. First, image augmentation techniques are adopted to increase the sample size of the sheep thoracic vertebrae and scapulae to overcome the problems of long-tailed distribution and non-equilibrium of the dataset. Then, the performances of three structural variants of the Swin-Transformer (Swin-T, Swin-B, and Swin-S) are compared through transfer learning, and the optimal model is obtained. On this basis, the robustness, generalization, and anti-occlusion abilities of the model are tested and analyzed using the significant multiscale features of the lumbar vertebrae and thoracic vertebrae, by simulating different lighting environments and occlusion scenarios, respectively. Furthermore, the model is compared with five methods commonly used in object detection tasks, namely Sparser-CNN, YoloV5, RetinaNet, CenterNet, and HRNet, and its real-time performance is tested under the following pixel resolutions: 576 × 576, 672 × 672, and 768 × 768. The results show that the proposed method achieves a mean average precision (mAP) of 0.943, while the mAP for the robustness, generalization, and anti-occlusion tests are 0.913, 0.857, and 0.845, respectively. Moreover, the model outperforms the five aforementioned methods, with mAP values that are higher by 0.009, 0.027, 0.041, 0.050, and 0.113, respectively. The average processing time of a single image with this model is 0.25 s, which meets the production line requirements. In summary, this study presents an efficient and intelligent mutton multi-part classification and detection method, which can provide technical support for the automatic sorting of mutton as well as for the processing of other livestock meat.

## 1. Introduction

Mutton is an important part of the human daily diet structure, and the production of mutton has been increasing annually. According to statistics, China’s mutton production was expected to reach 5.14 million tons in 2021, up 4.4% from 2020 [1]. Mutton multi-parts, which are the final form of mutton products before marketing, are generally obtained by livestock meat processing enterprises on the basis of different meat cut standards for cutting sheep carcasses. These multi-parts are mainly categorized into two major groups: four-parts and six-parts [2,3,4]. Currently, Chinese mutton processing enterprises mainly use semi-automatic processing technology in the mutton multi-part sorting process, where manual sorting and stacking of the multi-part mutton is performed on a conveyor belt. This approach is highly labor intensive and involves a poor working environment as well as food safety hazards due to human–animal cross-infection [5]. Therefore, it is important to develop automatic sorting technology for mutton multi-parts in order to enhance the competitiveness of the industry. However, achieving this goal requires automatic, fast, and accurate extraction of mutton multi-part category information; hence, it is necessary to study the automatic classification and detection of mutton multi-parts.

Common technical methods used for mutton detection in recent years mainly include image processing, spectroscopic techniques combined with machine learning classification, and regressors. By extracting and analyzing multi-dimensional feature information such as color, texture, contour, protein, water content, and total volatile basic nitrogen (TVB-N) in mutton sample images, and establishing the relationship with mutton freshness [6,7,8], tenderness [9,10,11], authenticity [12,13], pH [14,15], storage time [16,17], and other indicators, these methods allow effective and nondestructive detection of mutton quality. Although the aforementioned technical methods can achieve high detection accuracy, they also have shortcomings such as cumbersome artificial extraction of sample features, poor generalization of models, and low adaptability, which are not suitable for the classification and detection of mutton with multiple categories, large quantities, and complex natural feature expression.

The computer vision object detection method based on deep learning, which has strong independent extraction, learning, and reasoning capabilities for deep and shallow features of sample images, can better solve the aforementioned problems. Zhao et al. [18] generated sheep skeleton images using generative adversarial networks and conducted image semantic segmentation research based on ICNet for the key parts of the sheep skeleton in various scenes; they achieved an average segmentation accuracy of >90%. Meng et al. [19] used image processing technology combined with the back propagation (BP) neural network method to distinguish the sheep back, foreleg, and hindleg meat under different storage time gradients. Zhang et al. [20] developed a non-contact measurement system for sheep shape parameters using machine vision technology, and thus solved the problem of measuring the sheep body size parameters under non-stress conditions. Liu et al. [21] proposed a method for recognizing the muscle area of the sheep hind leg using R2U-NET, and achieved an average accuracy of 0.982. Wang et al. [22] developed a sheep carcass segmentation method based on surface convexity by scanning and processing 3D point cloud information. Zhao et al. [3] used YoloV3 to realize accurate recognition of multiple mutton parts; however, the detection accuracy requires further improvement. The aforementioned research methods and results can be used as a reference and explored further to achieve high-precision classification and detection of multiple mutton parts.

In 2017, Vaswani et al. [23] proposed the Transformer network, which has shown excellent performance in the field of natural language processing. Through further studies, researchers have made several improvements to the Transformer, resulting in networks such as DETR [24], ViT [25], and SETR-MLP [26]. These networks have been applied to various fields, such as object detection [27,28,29], semantic segmentation [30,31,32], image classification [33,34,35], and image generation [36]. In this study, the advanced Swin-Transformer [37] network is used to conduct research in the following three aspects: (1) Proposing a high-precision classification and detection model for mutton multi-parts; (2) testing the robustness, generalization, and anti-occlusion performance of the proposed model; (3) introducing other mainstream detection algorithms to evaluate the advantages and disadvantages of the proposed model and test its real-time performance. The proposed classification and detection method for mutton multi-parts can provide new technologies and ideas for automatic and intelligent processing of mutton products. It can also serve as a technical reference for automatic classification and sorting of other livestock meat.

## 2. Materials and Methods

### 2.1. Test Materials and Image Acquisition

**Figure 1:**
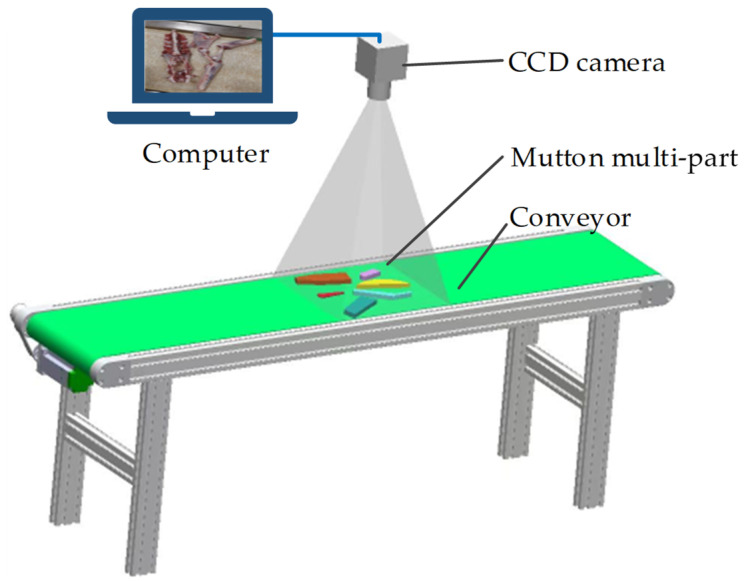
Mutton multi-part images acquisition device.

**Figure 2:**
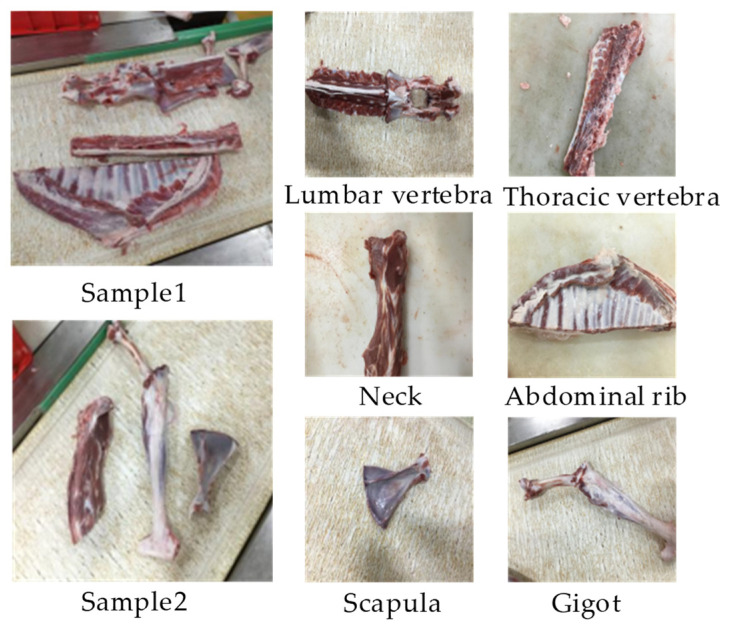
Sample of mutton multi-part images.

We used six types of mutton parts, namely the lumbar vertebrae, thoracic vertebrae, neck, abdominal ribs, scapulae, and gigot, obtained from adult Boer goats with a breeding-age of 12 months or older after slaughter as test materials. The sample images were collected from the sheep carcass cutting workshop scene of Meiyang Food Co., Ltd., Bayannur City, Inner Mongolia Autonomous Region. During the mutton multi-part image acquisition process, the mutton parts were randomly scattered on a 1.2 m-wide conveyor belt. A CCD camera (model WP-UC600, HuaGu Power Technology, Shenzhen, China) with an Omron Z4S-LE-SV-1214H lens was installed at a height of 0.6 m directly above the mutton parts, without any special light source or background. The layout of the image acquisition device is shown in Figure 1. Finally, mutton multi-part images with a resolution of 2448 × 3264 pixels were obtained. An example of the sample image is shown in Figure 2.

### 2.2. Sample Pretreatment and Establish Dataset

The accuracy of computer vision models based on deep learning is strongly correlated with the size of the dataset. A larger dataset is beneficial for improving the extraction and learning ability of the depth network for object features [38]. However, in this study, the number of sheep thoracic vertebrae and scapulae is far lower than that of other mutton parts. Consequently, the mutton multi-part image data have the characteristics of long-tailed distribution and non-equilibrium. It is necessary to expand the data of the sheep thoracic vertebrae and scapulae.

**Figure 3:**
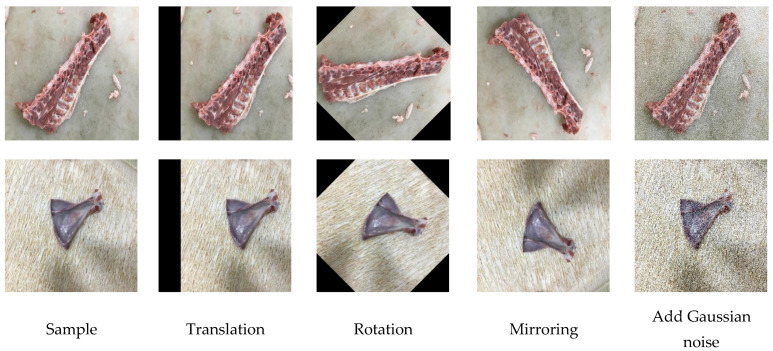
Enhancement of images of thoracic vertebra and scapula in mutton.

For this purpose, four image morphological operations were performed in this study: translating 100 pixels along the x-axis, rotating 45° clockwise, mirroring, and adding Gaussian noise with a mean value of 0 and variance of 0.2. These operations are shown in Figure 3.

After realizing image data augmentation of the sheep thoracic vertebrae and scapulae, a total of 12,000 mutton multi-part images were obtained. To reduce the complexity of the model training, the image size was scaled to 512 × 512 pixels in order to establish the mutton multi-part image dataset. Then, the dataset was divided into the training set, test set, and validation in the ratio of 8:1.5:0.5. The LabelImg image annotation toolbox and the VOC dataset format were employed to label the lumbar vertebrae, thoracic vertebrae, neck, abdominal ribs, scapulae, and gigot of sheep as Sheep1, Sheep2, Sheep3, Sheep4, Sheep5, and Sheep6, respectively. The distribution of the number of each part of the mutton multi-part image dataset was determined manually, as indicated in Table 1.

### 2.3. Principle of Swin-Transformer Algorithm

#### 2.3.1. Swin-Transformer Network Structure

**Figure 4:**
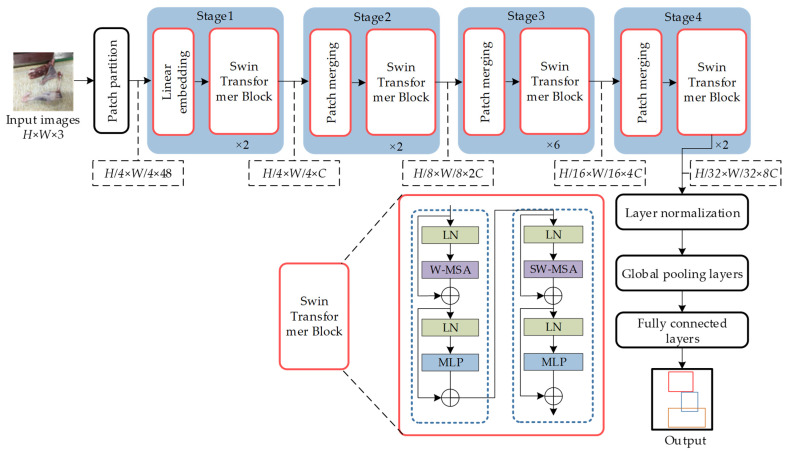
Swin-Transformer network structure. Note: H is the height of the input image, pixels; W is the width of the input image, pixels; C is the dimension of feature graph; LN is the Layer Norm; W-MSA is the Window Multi-head Self-Attention structure; SW-MSA is the Shifted Window Muti-head Self-Attention structure; MLP is Multilayer Perceptron; Stage1–Stage4 is cascading modules.

The network is designed on the basis of the shifted window operation, attention mechanism, and layering. It mainly consists of multi-layer perceptron (MLP), window multi-head self-attention mechanism (W-MSA), shifted window multi-head self-attention mechanism (SW-MSA), and layer normalization (LN), and it has the advantages of strong feature extraction ability, high prediction accuracy, fast reasoning, and a lower computational requirement compared to the original Transformer [39,40]. The structure of the Swin-Transformer network is shown in Figure 4.

First, the RGB three-channel mutton multi-part image with a size of 512 × 512 pixels is input to the Patch partition. Then, the Patch partition is chunked on the basis of the benchmark of each adjacent 4 × 4 pixel size and spread in the channel direction, resulting in a mutton multi-part image size of 128 × 128 × 48, which is subsequently input into Stage1. Stage1 consists of the Linear Embedding and Swin-Transformer blocks. The Linear Embedding block projects the original features of each image block into C = 128 dimensions to obtain a feature map of size 128 × 128 × 128, which is then transmitted to the Swin-Transformer block. The Swin-Transformer block contains residual connections, and performs the W-MSA attention calculation and the SW-MSA operation to improve the feature extraction efficiency while reducing network computation. In addition, the MLP is a two-layer perceptron with the Gaussian error linear unit (GELU) nonlinear activation function, and the W-MSA and SW-MSA preorders are arranged with LN. After the feature extraction and calculation in Stage1, the feature map is input into Stage2–Stage4, which contain Patch Merging and different numbers of Swing Transformer blocks. Patch Merging is used for reducing the resolution of the feature map by down-sampling it two times and merging it in the depth direction to expand the number of channels, and to form a layering design. Therefore, the resolution of the feature map decreases sequentially during the transfer process from Stage2 to Stage4, from 128 × 128 to 64 × 64 and 32 × 32, and the number of channels is adjusted from 128 to 256 and 512, respectively. Finally, Stage4 outputs the feature map with a size of 32 × 32 × 512, and the results of each mutton part category in the mutton multi-part image are obtained by LN, Global Pooling layers, and Fully Connected layers.

#### 2.3.2. Swin-Transformer Attention Mechanisms

**Figure 5:**
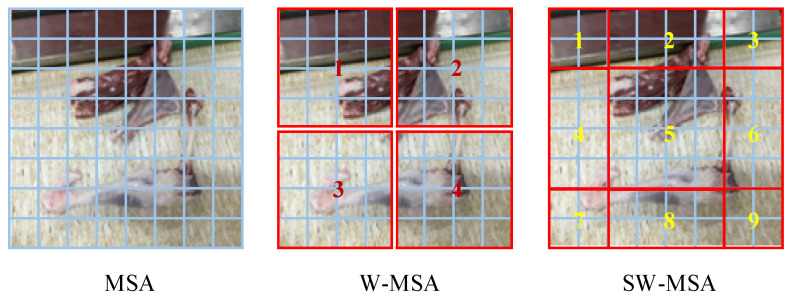
W-MSA and SW-MSA window division. Note: MSA is the multi-head self-attention mechanism. Note: MSA is the Multi-head Self-Attention structure; W-MSA is the Window Multi-head Self-Attention structure; SW-MSA is the Shifted Window Muti-head Self-Attention structure.

**Figure 6:**
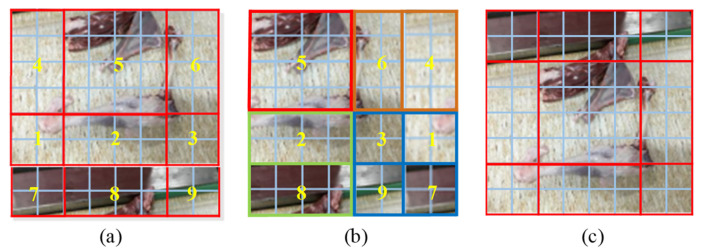
Swin-Transformer SW-MSA Shift configuration batch calculations. Note: (**a**–**c**) represent the three stages of the Shift configuration batch calculations process in Shifted Window Muti-head Self-Attention structure, respectively.

Although incorporating the MSA mechanism into the network structure can enhance the extraction effect of the object features and improve the detection accuracy, the retrieval of each pixel of the feature map by MSA significantly increases the computational complexity of the network, which is not conducive to network convergence [41]. To address this issue, the Swin-Transformer network uses two types of MSA: W-MSA and SW-MSA. W-MSA divides the feature map into windows with a fixed size of M × M (M = 4) and calculates the self-attention of each window. However, there is a lack of information exchange between non-overlapping windows, and there is a likelihood of errors in extracting object features distributed between different windows. Therefore, SW-MSA uses a shifted window partition to expand the receptive field and solve the problem of information acquisition of non-overlapping windows. This approach expands the four windows of W-MSA to nine windows. Figure 5 shows the specific window division. After the subdivision of the window by SW-MSA, the window size is different, necessitating window configuration adjustments. The first row of the window in Figure 5 SW-MSA is shifted to the third row (Figure 6a), and the third row of the window is then swapped with the second row (Figure 6b). This completes the shifted splicing of the windows from 3 × 3 to 2 × 2. Finally, after the MSA calculation of the new window, the data are moved back to the original position, as shown in Figure 6c.

The Swin-Transformer based on W-MSA uses a window of size M × M as the unit to calculate the image area, and the network computational complexity is significantly reduced compared with the MSA with a block as the computational unit. Assuming that the image size is h × w and C is the feature map dimension, the computational complexities of MSA and W-MSA are respectively given by Equations (1) and (2).

(1) $$\Omega (\mathrm{MSA})=4hwC^{2}+2{\left(hw\right)}^{2}C$$

(2) $$\Omega (\mathrm{W-MSA})=4hwC^{2}+2M^{2}hwC$$

As can be seen from Equations (1) and (2), the computational complexity of W-MSA is linearly related with the size of the image, while the computational complexity of MSA has a quadratic power relationship. With the same image size, the number of windows is significantly smaller than the number of blocks, resulting in a significant reduction in the computational complexity.

### 2.4. Evaluation Index

In this study, the mean average precision (mAP) commonly used in object detection tasks is employed as an indicator to quantify the performance of the mutton multi-part classification detection model. It characterizes the average model detection accuracy for six types of mutton parts. A larger value indicates better detection performance. In addition, the AP is the detection accuracy of the same model for a certain category of mutton multi-parts, which is obtained from the integration of the precision–recall (P−R) curve. The precision P, recall R, and AP are calculated as shown in Equations (3) and (4):

(3) $$\left\{\begin{array}{c} P=\frac{TP}{TP+FP}\\ R=\frac{TP}{TP+FN} \end{array}\right.$$

In Equation (3), TP and FP respectively denote the number of positive samples correctly judged and the number of positive samples wrongly judged, while FN denotes the number of negative samples wrongly judged.

(4) $$\left\{\begin{array}{c} AP=\int_{0}^{1}PRdr\\ R=\frac{1}{M}\sum_{K-1}^{M}AP(K) \end{array}\right.$$

In Equation (4), M denotes the number of all categories; in this study, M is 6. Further, AP(k) denotes the detection accuracy of category k objects; in this study, the value range of k is 1–6.

Moreover, the average speed of the model for quantitative mutton multi-part image processing is obtained as an index for judging the real-time performance of the model detection, which is obtained via multiple repetitions of the test followed by averaging. A smaller value indicates better real-time performance.

### 2.5. Method of Adjusting Image Brightness

To simulate the acquisition of mutton multi-part images under different lighting conditions, we used the OpenCV image processing function library in order to convert the color space of the mutton parts image to HSV. Then, the V channel value representing the image brightness was used to “lighten” and “darken” the image in a multiple relationship of 1.35 and 0.75, respectively. Finally, the image was converted back to the original RGB color space. The relationship between image brightness and the value of the V channel is shown in Equation (5).

(5) $$\left\{\begin{array}{c} lighten=V\times 1.35\\ darken=V\times 0.75 \end{array}\right.$$

### 2.6. Test Environment

The mutton multi-part classification and detection test was conducted using a customized Lenovo computer equipped with an AMD Ryzen 5 CPU operating at a dominant frequency of 3.90 GHz, 16 GB of operating memory, and an NVIDIA RTX3060 GPU with 12 GB of graphics memory. The operating system was Windows 11, and a virtual environment was established using the Python 3.8 programming language to conduct model training and performance measurements based on the PyTorch 1.8.0 deep learning framework. For GPU-accelerated computing, cuDNN 8.1.1 was used with DUDA version 11.0.

## 3. Experiment and Results

**Figure 7:**
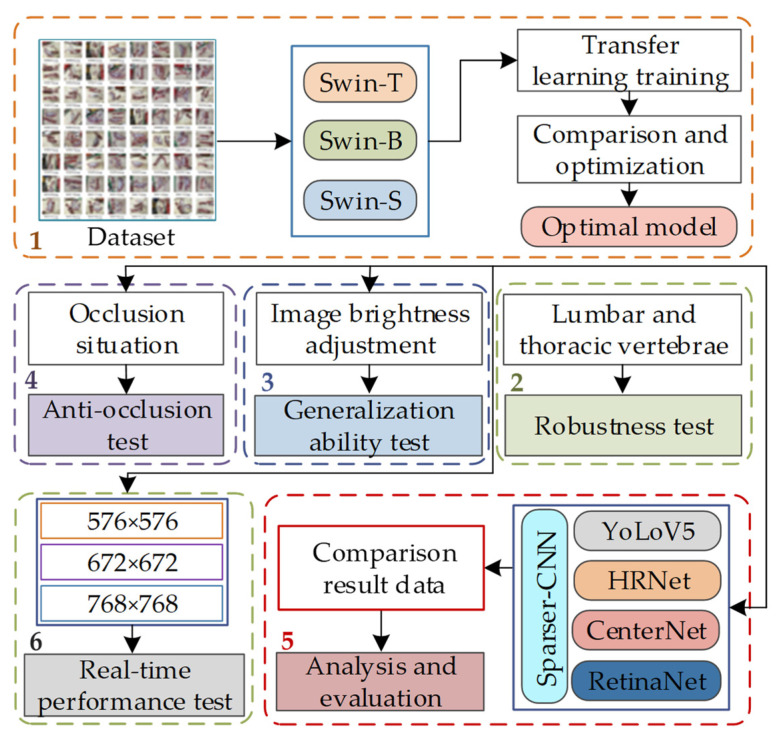
Test flow of classification and detection of mutton multi-part based on Swin-Transformer. Note: 1 is the optimal model acquisition experiment; 2 is the robustness test; 3 is the generalization ability test; 4 is the occlusion resistance test; 5 is the performance comparison experiment; 6 is the real-time performance test.

The mutton multi-part classification and detection test using the Swin-Transformer network consists of six steps as follows:

1. For the mutton multi-part image dataset, three types of Swin-Transformer network, namely Swin-T, Swin-B, and Swin-S, are introduced to conduct model training and comparison, and obtain the optimal mutton multi-part classification and detection model.
2. The generalization ability of the model is tested for the lumbar vertebrae and thoracic vertebrae of mutton with obvious multi-scale features.
3. The generalization ability of the model is tested by adjusting the brightness of the mutton multi-part image.
4. Mutton multi-part images with occlusion scenarios are selected to test the anti-occlusion ability of the model.
5. Other common object detection algorithms are introduced for comparative analysis in order to judge the advantages and disadvantages of the model.
6. The image resolution is adjusted and the real-time performance of the model is tested. The test flow is shown in Figure 7.

### 3.1. Classification and Detection of Mutton Multi-Part Based on Swin-Transformer

From the Swin Transformer, Swin-T, Swin-B, and Swin-S were derived by adjusting the feature map dimension and network structure. To obtain the best model, this study selected the three aforementioned Swin Transformer network variants as backbone networks and introduced Mask-RCNN for comparative testing. The network parameters of Swin-T, Swin-B, and Swin-S are summarized in Table 2.

**Figure 8:**
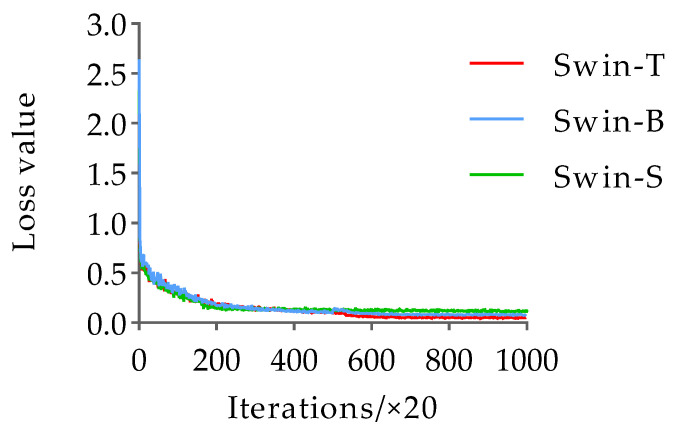
The loss value changes with the number of iterations.

**Figure 9:**
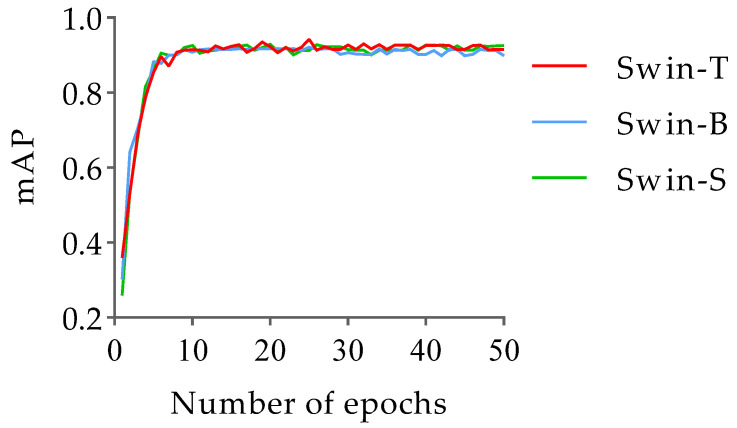
The mAP value changes with the number of iterations.

In this study, pre-trained weights for ImageNet datasets were loaded into the Swin-T, Swin-B, and Swin-S networks for training in order to prevent overfitting and accelerate network convergence. The test employed stochastic gradient descent (SGD) for gradient descent, with the momentum and weight decay set to 0.9 and 0.0001, respectively. Further, the learning rate and batch size were set to 0.002 and 16, respectively. In addition, the model training followed the strategy of saving the loss value once every 10 iterations and saving the model once every epoch, with a total of 20,000 iterations and 50 epochs. For the mutton multi-part image dataset, the change trends of the loss and mAP values with the number of iterations during the training of the Swin-T, Swin-B, and Swin-S networks are shown in Figure 8 and Figure 9.

**Figure 10:**
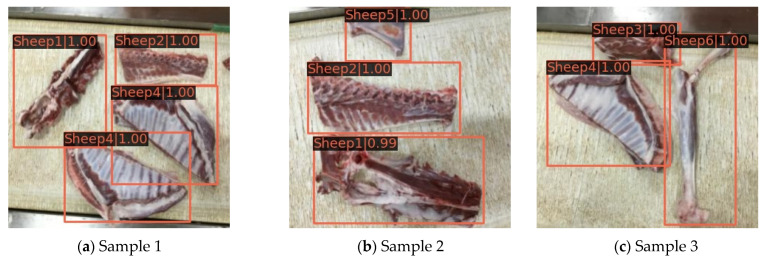
Partial detection results of the model based on Swin-T mutton multi-parts classification detection.

From Figure 8, it can be seen that the loss values of the Swin-T, Swin-B, and Swin-S networks all decreased rapidly in the early stage of training, until the number of iterations was around 2000, when the loss values of the three networks start to smooth; then, the loss values of the Swin-S network gradually converged to 0.123, while at this time, the loss values of the Swin-T and Swin-B networks still had a slight decreasing trend. The Swin-B network training converged to a loss value of 0.081 when the number of iterations reached 5500, while the Swin-T network loss value continued to decline until 6500 iterations, and it gradually converged to 0.054. In addition, according to the change trend of the mAP value of the model training with the number of epochs in Figure 9, it can be seen that the changes in the mAP value of each network during the training of the Swin-T, Swin-B, and Swin-S networks were basically the same; they all increased substantially in the initial stage of training, and then fluctuated slightly after 10 epochs until the training ended. On this basis, when the number of epochs was 25, the mAP value of the Swin-T network was the highest, reaching 0.943, while the mAP values of the Swin-B and Swin-S networks were the highest at 0.927 and 0.931, which were 0.016 and 0.012 lower than those of Swin-T, respectively. Based on these results, the classification and detection model of mutton multi-part obtained on the basis of the Swin-T network at epoch = 25 has the highest detection accuracy and the best classification performance. Therefore, this model was selected for the classification and detection of mutton multi-parts as well as for all the subsequent performance tests. The partial recognition results of the model for the mutton multi-part image validation set are shown in Figure 10.

**Figure 11:**
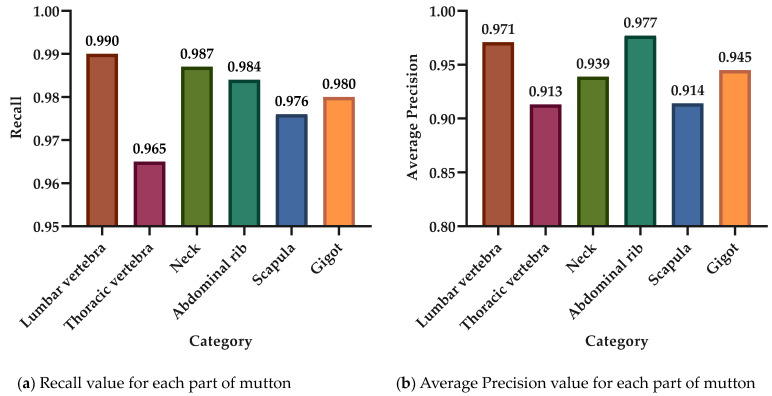
The Recall and AP of mutton parts based on Swin-T mutton multi-parts classification detection model.

Under the background of the current mutton processing technology, the mutton parts fall on the conveyor belt in a periodic and intermittent manner, forming a region, which is manifested as a single image containing multiple, multi-class, and repeated mutton parts, as shown in Figure 10. In Figure 10a–c, the detection of various types of mutton parts does not appear to be incomplete or incorrect; all the categories of each mutton component can be detected with a high degree of confidence. Meanwhile, the bounding box marking area is correct and complete, especially the relatively small scapula in sample 2 and the lumbar vertebrae and thoracic vertebrae in samples 1 and 2 with large differences in shape; the model could accurately detect them, indicating that it could achieve accurate classification detection of various mutton parts. Finally, the Recall and AP of the six types of mutton parts obtained on the basis of the Swin-T mutton multi-part classification detection model are shown in Figure 11a,b.

From Figure 11, it is evident that the Recall for the thoracic vertebra is the lowest, while that for the lumbar vertebra is the highest. However, the difference between them is not significant, and both can exceed 0.950. Moreover, the AP value of the various mutton parts is consistent with the change in Recall, and the AP value of all the mutton parts obtained is greater than 0.900. Ultimately, combined with the results in Figure 10, we can infer that the model has a remarkable ability to classify and detect various mutton parts.

### 3.2. Robustness Testing

**Figure 12:**
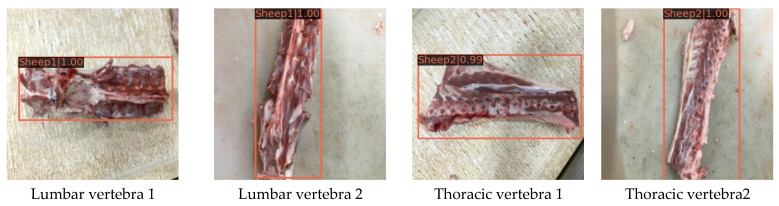
Partial results of model robustness testing in this paper.

To evaluate the ability of the proposed model to detect the mutton parts with large differences in size, we selected the lumbar and thoracic vertebrae of sheep with significant multi-scale characteristics as samples in order to test the robustness of the model. Based on the large-scale difference between the samples, we manually screened 100 lumbar vertebrae and thoracic vertebrae and collected the corresponding images to establish the robustness test dataset. Subsequently, we conducted the robustness test using the proposed model. Some results are shown in Figure 12.

From Figure 12, it is evident that the same category of lumbar vertebra and thoracic vertebra exhibit significant differences in size and shape. The aforementioned multi-scale characteristics lead to difficulties in the accurate detection of some mutton multi-parts. However, according to the recognition results in Figure 12, the proposed mutton multi-part classification and detection model can effectively exclude the aforementioned recognition interference due to scale differences and correctly infer the sample category and location with a high confidence level and small bounding box error. Finally, the mAP value of the proposed model for the robustness test dataset reached 0.913; thus, the model has good robustness for mutton multi-part classification detection with obvious multi-scale characteristics.

### 3.3. Generalization Testing

**Figure 13:**
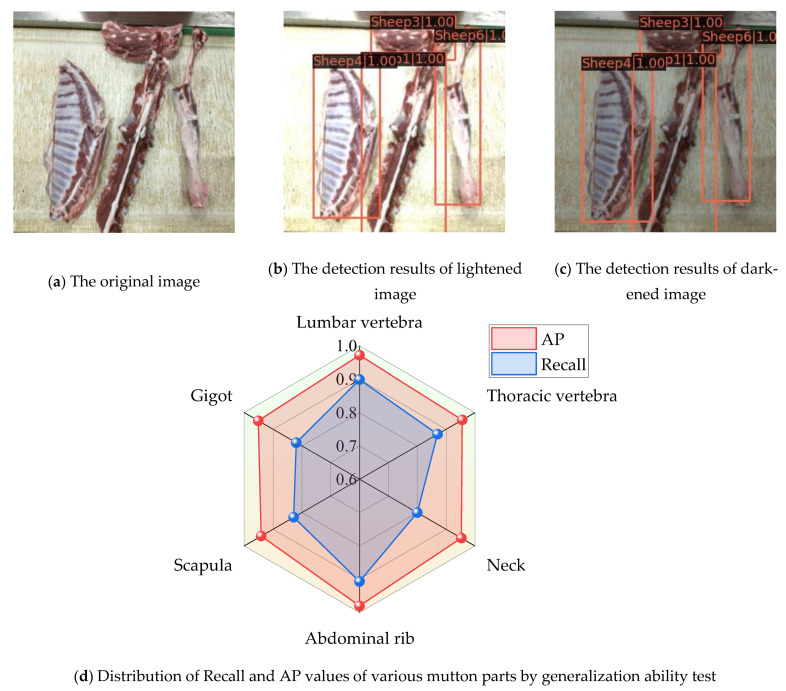
Partial results of the generalization ability test of mutton multi-parts classification and detection.

The mutton multi-part images were acquired under consistent energy-saving lighting conditions in the production workshop. Although the proposed model can achieve higher detection accuracy in these scenes, its performance under different lighting conditions is uncertain. To this end, we randomly selected 200 mutton multi-part images, simulated different lighting conditions by adjusting the brightness level of each image, and finally established a generalization ability test dataset comprising a total of 400 images. With regard to the generalization ability test dataset, the partial test results of the model generalization ability test and the Recall, AP values of various mutton parts are shown in Figure 13a–c.

From the detection results in Figure 13b,c after the brightening and darkening of the mutton multi-part images, we can see that the proposed model can accurately detect multiple types of mutton parts in different light intensity scenes with high confidence; at the same time, there is no obvious error between the real contour of the mutton parts and the marked bounding box. Combined with the radar plots of the Recall and AP values for various types of mutton part detection in Figure 13d, the Recall values of the six types of mutton are all distributed in the range of 0.9–1.0, and the AP values are all >0.8. Finally, for the generalization ability test data set, the mAP value of the proposed model reaches 0.857, indicating that it can be applied to the classification and detection of mutton multi-parts in different brightness scenes, and it has strong generalization ability.

### 3.4. Anti-Occlusion Testing

**Figure 14:**
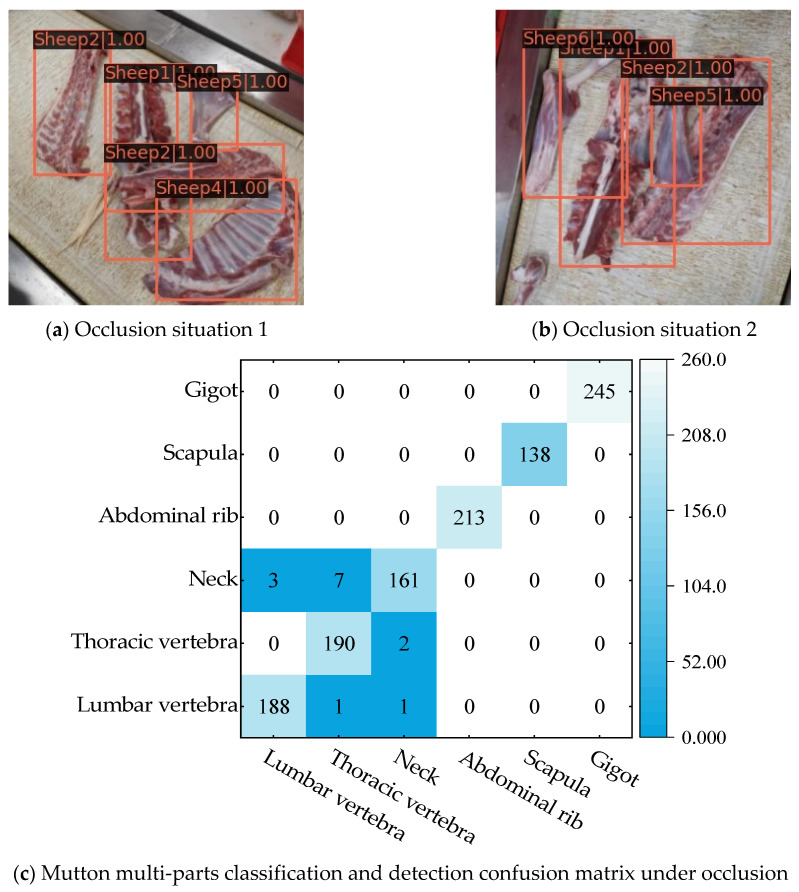
Partial results of the mutton multi-parts classification and detection under occlusion.

The mutton multi-parts are randomly scattered on the conveyor belt, which causes a small amount of stacking, blocking, and feature loss. However, the mutton multi-parts in the blocked state may belong to the same category. The aforementioned intersection of mutton multi-part regions, feature loss, and similar situations make it difficult to accurately classify and detect mutton multi-parts. However, the unobscured part of the mutton multi-part image retains mutually independent features; if the model can accurately identify the features and distinguish them correctly, it can still achieve correct classification detection of mutton multi-parts. Considering the occlusion status of the mutton parts, 200 images of mutton multi-parts with occlusion were selected to establish the occlusion dataset in order to test the anti-occlusion ability of the model. Some test results are shown in Figure 14.

In Figure 14a,b, the thoracic vertebrae and lumbar vertebrae are partially occluded, and the scapula and gigot are adhered with similar features at the same time, which may easily result in the thoracic vertebrae occluded parts being wrongly detected as lumbar vertebrae, as well as incomplete or incorrect detection of the scapula. However, the proposed model can still accurately detect the category and position of the corresponding mutton parts in the image for the aforementioned situation, because the occluded area of the thoracic vertebrae and lumbar vertebrae is relatively small compared with the remaining area, and they still retain a large number of natural features; this makes the occluded area have a smaller impact on the object category inference and bounding box positioning of the model. In addition, the window multi-head self-attention mechanism and shifted window multi-head attention mechanism in the Swin-Transformer network structure improve the ability of the model to extract and learn the semantic information of the image context, thereby making it easier for the model to distinguish edge contours under the condition that the mutton multi-parts adhere to each other and the features are similar; this reduces the probability of false or incomplete detection. Finally, for the occlusion dataset, the detection results of the present mode for all types of mutton parts are shown in the confusion matrix of Figure 14c. Except for three of Lumbar vertebra, eight of Thoracic vertebra, and three of Neck, which were detected by mistake, all the other mutton parts were detected accurately, and the average detection accuracy of the model reached 0.845, indicating that the model has strong anti-occlusion ability and can accurately identify the category and position of the occluded mutton parts.

### 3.5. Comparison and Analysis with Other Methods

**Figure 15:**
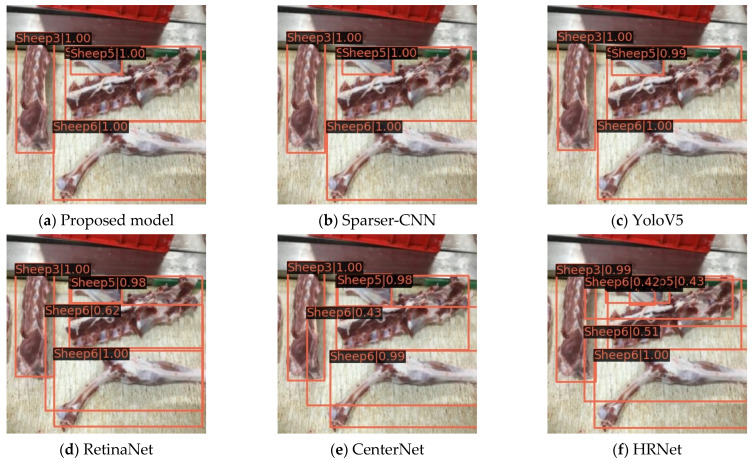
Recognition results of proposed model compared with others models.

Owing to the development of deep learning technology, object detection methods are becoming increasingly diverse and the detection performance is being continuously improved; however, the performances of different methods in the case of different object detection tasks are often different [42]. To explore the advantages and disadvantages of the proposed Swin-T mutton multi-part classification detection model compared to mainstream object detection methods, we introduced Sparser-CNN [43], YoloV5 [44], RetinaNet [45], CenterNet [46], and HRNet [47] in order to conduct comparison tests on the mutton multi-part image datasets. During the experiment, the computer platform, compilation environment, and training hyperparameters used were consistent with those used for the Swin-Transformer. The partial recognition results of the five aforementioned methods for each type of mutton part in the mutton multi-part image validation set are shown in Figure 15.

**Figure 16:**
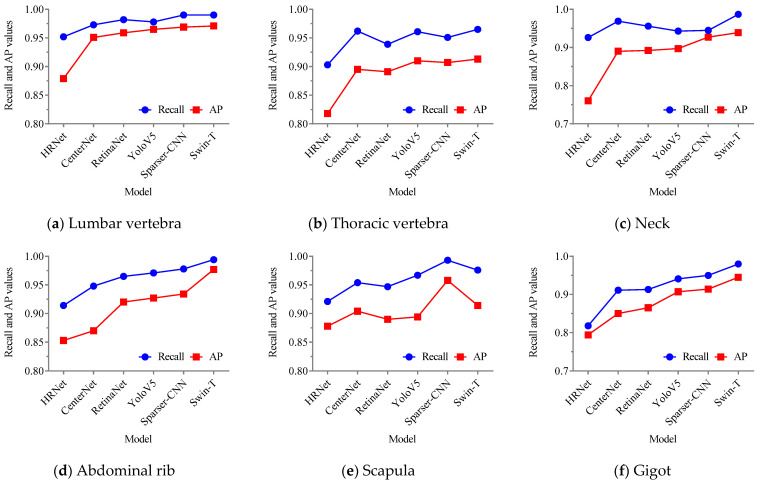
Comparison of Recall and AP values of each mutton parts obtained by the proposed model and other models for the validation set.

From the results in Figure 15a,b, it can be seen that the category and bounding box localization of all types of mutton parts are accurately detected without false negatives, and the bounding box is accurately tangent to the object region without obvious pixel errors; thus, the proposed model has excellent performance, which is similar to Sparser-CNN. By contrast, the YoloV5 detection results in Figure 15c show that the various mutton part categories and locations are correctly distinguished; however, the confidence level of the scapulae category is lower than that in Figure 15a,b, indicating that although the YoloV5 detection performance is excellent for mutton multi-parts, it is weaker than that of the proposed model and Sparser-CNN. In addition, in Figure 16d–f, the detection of the gigot was false, i.e., the single gigot was detected as multiple gigots, and the confidence of the scapula in Figure 15f was only 0.43, indicating that under this test environment, RetinaNet and CenterNet had similar performance, while HRNet had the weakest detection ability; none of them was suitable for the mutton multi-part classification and detection. Moreover, the Recall and AP values for each method were obtained to quantify the differences between the methods. The results of the comparison of the Recall and AP values for each mutton part by the different methods are shown in Figure 16.

From Figure 16, there is a difference of around 0.200 in the classification detection accuracy of the different models for different mutton parts, and they all have high detection accuracy for the lumbar vertebra and abdominal rib, and low detection accuracy for the neck and gigot. In addition, through comparison, it is found that among the five types of mutton part classification detection except scapula, the proposed mutton multi-part classification detection model has the highest detection accuracy, Sparser-CNN is the second best, followed by YoloV5, which is better than RetinaNet and CenterNet; HRNet has the weakest performance. The reasons may be as follows: (1) The scapula is a small object sample among the six types of mutton parts in this study; its size is smaller than that of the other mutton parts, while its color characteristics are similar to the background of the conveyor belt and the contrast is low, which is not conducive to comparison and unification for obtaining its classification confidence and bounding box positioning. (2) The Swin-Transformer network has multiple down-sampling, a layering structure, a cascaded multi-dimensional feature map, and a shifted window mechanism; thus, it has better feature extraction, learning and inference capabilities than ordinary CNN, and it allows refining of the local context semantic information of the image, which is beneficial for the classification detection of mutton multi-parts in the state of adhesion and occlusion. (3) Sparser-CNN first generates region proposals for the image and then performs classification and regression on these region proposals. By contrast, YoloV5, RetinaNet, and CenterNet only perform regression operations on the image. Thus, Sparser-CNN has higher detection accuracy than other methods when detecting small objects such as the scapula. (4) The research object has the characteristics of similar natural feature expression, while the strategy of HRNet of gradually reducing the image resolution followed by continuous up-sampling to improve the resolution of the feature map can easily cause feature loss and confusion in the mutton multi-parts with similar features, which makes the detection accuracy lower than that of other models.

**Figure 17:**
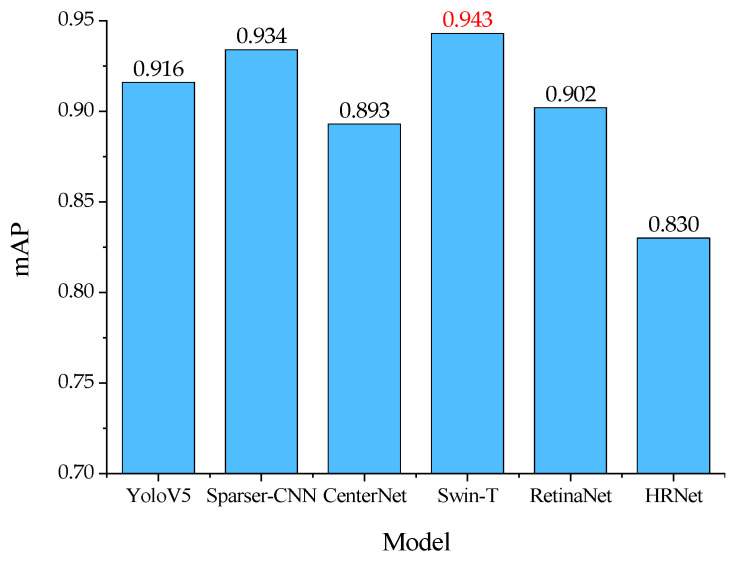
Comparison of mAP value by the proposed model and other models for the validation set.

Figure 17 shows the comparison results of the mAP of the mutton multi-part classification and detection model-based Swin-transformer proposed in this paper with YoloV5, Sparser-CNN, CenterNet, RetinaNet, and HRNet for mutton segmentation image validation set. It can be seen that the mAP value of proposed model is 0.027, 0.009, 0.050, 0.041, and 0.113 higher than that of the aforementioned five object detection methods, respectively, and the detection performance is the best.

### 3.6. Real-Time Performance Test

To further explore the real-time performance of the proposed model in practical application, the original image resolution is divided into three levels: 576 × 576, 672 × 672, and 768 × 768 pixels. The number of images at each resolution was set to 300, and the real-time performance of the model was tested for processing images of different pixel sizes. The test was repeated three times for each resolution image; then, the average total detection time taken for each resolution image and the average processing time of a single image were calculated. The average processing time of a single image was regarded as the final indicator to judge the real-time performance of the model. A smaller value indicates better real-time performance; conversely, a larger value indicates worse real-time performance. The results of the statistical analysis are summarized in Table 3.

According to the results in Table 3, the total detection time of the proposed model increases with the image resolution, leading to a corresponding increase in the processing time of a single image. This could be due to the gradual increase in the floating-point operations required for the model calculations as the image resolution increases, thereby increasing the classification and detection reasoning time. However, the proposed model has an average processing time of 0.25 s for a single image, indicating that it still maintains good real-time performance and is suitable for real-time detection applications in mutton food-processing production lines.

## 4. Discussion

To address the need for further classification and detection of mutton multi-parts in the conveyor belt scenario, this paper proposed an automatic and accurate detection method of mutton multi-parts based on computer vision technology. Owing to the characteristics of mutton parts with similar features and large multi-scale differences, as well as the brightness change in actual production scenes, mutual occlusion, and the need for higher production speed, a classification detection method with robustness, generalization, and anti-occlusion abilities as well as real-time performance is required. We verified the excellent comprehensive detection ability of the proposed method through multiple targeted tests and comparative tests.

The current research on the recognition and detection of mutton parts basically adopts image processing combined with machine learning and deep learning. The techniques used, research subjects, and obtained results of the methods are summarized in Table 4.

Compared with related studies, the proposed model corresponds to the most mutton categories, and has the highest classification detection accuracy. Furthermore, the proposed method has several important advantages. For example, in the case of complex and similar mutton parts, it does not rely on manual selection of multi-dimensional features of mutton multi-parts for extraction, as well as the same good detection effect in the brightness changes, occlusion situations. Therefore, it is applicable to various scenarios. However, the main disadvantage is that it is limited by the body structure of the sheep carcass and the processing technology; the number of the sheep lumbar spine and scapula is small, which is not conducive to the improvement of the model classification and detection accuracy, and manual data expansion is required. Moreover, when mutton food processing enterprises adopt different processing technologies, the types of mutton multi-parts are also different, including four-parts in addition to the six-parts in this study, for which the method has not been verified.

Overall, the research results show that the proposed classification and detection method of mutton multi-parts can address the classification and detection of mutton parts for various situations on the conveyor belt. However, the proposed method only verifies the static image, and it does not obtain the spatial distance information of the mutton parts. In the future, the image acquisition equipment could be replaced with RGB-D cameras to obtain the depth characteristics of mutton multi-parts. This could be applied to real-time video processing on the production line in order to improve the depth distance guidance for automatic sorting devices. Potential applications to other livestock meat processing could also be explored.

## 5. Conclusions

This paper proposed a classification and detection method for mutton multi-parts based on the Swin-Transformer. By comparing the performances of Swin-T, Swin-B, and Swin-S, the model based on Swin-T was found to have the highest mAP of 0.943, and it could accurately detect the category and position of multiple and multi-class mutton parts in the image. Moreover, the proposed method was verified to have excellent overall detection capability by testing its robustness, generalization, anti-occlusion abilities, and real-time performance, as well as performance comparisons with other methods. The proposed method has a certain reference significance for automatic sorting technology and equipment research of livestock meat, and it provides a certain foundation for the development of intelligent meat processing. Future research will focus on the visual weight measurement of mutton multi-parts and output estimation.

## Figures and Tables

**Table 1:** Distribution of the number of different types of parts in the mutton multi-part images dataset.

Category	Training Set	Test Set	Validation Set
Lumbar vertebrae	3127	935	412
Thoracic vertebrae	3283	1066	467
Neck	3015	1149	334
Abdominal ribs	4398	1439	538
Scapulae	2490	981	359
Gigot	5329	1862	564
Total	21,642	7432	2674

**Table 2:** Parameters of three Swin-Transformer network variants.

Network	Image Size (Pixel)	*C*	Number of Stage1	Number of Stage2	Number of Stage3	Number of Stage4
Swin-T	512 × 512	96	2	2	6	2
Swin-B	512 × 512	96	2	2	18	2
Swin-S	512 × 512	128	2	2	18	2

**Table 3:** Real-time test results of the proposed model under different image resolutions.

Image Resolution	Average Total Detection Time Consuming/s	Average Processing Time of Single Image/s
576 × 576	73.33	0.24
672 × 672	76.33	0.25
768 × 768	80.65	0.27
Mean	76.77	0.25

**Table 4:** Comparison of the proposed model with existing related work.

References	Test Objects	Category	Method	Result
Zhang et al. [17]	Lamb leg, Duck leg, Pork leg, and chicken breast	4	CNN	AP > 0.940
Zhao et al. [18]	Neck, Spine, and caudal vertebra	3	ICNet	MIoU = 0.858, 0.906, 0.757
Meng et al. [19]	Back meat, Hind Leg, and front Leg	3	Image Processing and BP	ACC = 0.914
Zhang et al. [20]	Sheep’s Conformation Parameters	4	Image Processing	σ < 6 cm
Liu et al. [21]	Muscle region in hind leg	1	R2U-NET	AP = 0.982
Wang et al. [22]	Sheep carcass	1	PCL	Average overall ACC = 0.921
Proposed model	Lumbar vertebrae, Thoracic vertebrae, Neck, Abdominal rib, Scapulae, and gigot	6	Swin-Transformer	mAP = 0.943

## Data Availability

Data is contained within the article.
